# Unearthing Influenza's Unusual Neurological Impact

**DOI:** 10.7759/cureus.73203

**Published:** 2024-11-07

**Authors:** Syed Zahan Raza, Tariq Masood, Muhammad Akram

**Affiliations:** 1 Acute Medicine, Northern Lincolnshire and Goole NHS Foundation Trust, Scunthorpe, GBR

**Keywords:** infection, influenza, polyneuropathy, respiratory, viral

## Abstract

Influenza is a viral infection primarily affecting the upper and lower respiratory tract. Beyond its respiratory manifestations, influenza is recognised for inducing certain neurological complications, including cognitive impairment, encephalopathy, and seizures. Studies have also shown an association between influenza infection, vaccination and Guillain-Barre Syndrome.

A middle-aged male patient initially manifested flu-like symptoms, followed by the development of peripheral neuropathy affecting peripheral nerves at different sites. Despite comprehensive investigations including cerebral imaging, lumbar puncture, infectious, vasculitis, HIV, and syphilis screening, no significant findings emerged. Viral PCR conclusively identified Influenza A infection; hence he was treated conservatively. The patient's symptoms demonstrated prompt and spontaneous recovery with supportive treatment alone, leading to a strong presumption that the neuropathy was attributable to the influenza A infection.

## Introduction

Influenza, commonly known as the "flu," is a highly contagious viral infection caused mainly by influenza viruses A and B. It primarily targets the upper respiratory tract, nose, throat, and bronchi, but can extend to the lungs, causing complications like pneumonia. In severe cases, influenza may affect other organs, such as the heart, brain, and muscles, leading to broader health complications.

Globally, influenza poses a significant health burden. According to the World Health Organisation (WHO), there are approximately one billion cases of seasonal influenza annually, including 3-5 million severe cases, resulting in 290,000-650,000 respiratory-related deaths. A notable 99% of influenza-related respiratory deaths among children under five occur in developing countries.

Transmission occurs mainly through respiratory droplets expelled when an infected person coughs, sneezes, or speaks, and the virus can also survive on surfaces, potentially spreading through contact. Symptoms typically appear 1-4 days post-infection and last about a week.

Influenza is also associated with neurological complications, such as seizures, cognitive impairment, and encephalopathy [1]. A recent case study on 1,277 children diagnosed with influenza showed that 131 (10.3%) were hospitalised for neurological symptoms, primarily seizures (73.3%) and encephalitis (29%) [2].

Research studies also link influenza infection and, in rare cases, vaccination to peripheral neuropathies like Guillain-Barré Syndrome (GBS) [3,4]. 

Here we report a case of influenza A infection complicated by peripheral neuropathy affecting multiple sites.

## Case presentation

A male patient in his 50s with no past medical history presented with flu-like symptoms, headache, fever and shortness of breath for three days. Over the period of the next six days, he developed generalised weakness in both upper and lower limbs with associated pins and needle sensation. Due to low oxygen stats, oxygen supplementation was commenced. Clinical examination revealed reduced power in both upper and lower limbs, MRC grading on 2-3/5 with left wrist drop & right foot drop. Reflexes were normal apart from planter reflex which were absent on both sides. Importantly, sensory functions remained unaffected. 

Comprehensive neuroimaging, including CT head, MRI head-spine, and lumbar puncture, yielded unremarkable results. Additionally, HIV, syphilis, vasculitis, infective and serum electrophoresis screening came back negative. Only influenza A virus was detected as shown in Table 1. A notable observation was made via electromyogram, revealing minor right common peroneal and left ulnar neuropathy, when patient symptoms were approaching near resolution. Importantly, no evidence of generalised neuropathy, specifically GBS, was discerned.

**Table 1:** Blood count

	Normal Range	Results
Sodium	133-146 mmol/L	143
Potassium	3.5-5.3 mmol/L	4.2
Chloride	95-108mmol/L	105
Urea	2.5-7.8 mmol/L	3.9
Creatinine	59-104 umol/L	81
Glomerular Filtration rate	90-200ml/L	>90
C-Reactive Protein	0-5mg/L	23
Haemoglobin	132-170g/L	130
White Cell Count	4.3-11.2 10*9/L	10.2
Platelets	150-400 10*9/L	288
Mean Corpuscular Volume	81-97 fL	82
Manual Neutrophil	2.1-7.4 10*9/L	8.29
Monocytes	0.3-1.0 10*9/L	1.01
Eosinophils	0.02-0.5 10*9/L	0.00
Vitamin B12	197-771ng/L	419
Serum Folate	2.0-18.7ug/L	9.9
Thyroid-Stimulating Hormone	0.27-4.5mu/L	1.2
Immunoglobulin G	7-16g/L	6.45
Immunoglobulin A	0.7-4.0g/L	1.16
Immunoglobulin M	0.4-2.3g/L	0.98
Serum Kappa	3.3-19.4mg/L	31.5
Serum Lambda	5.7-26.3mg/L	24.6
Serum K/L Ratio	0.26-1.65 ratio	1.28
Total Protein	60-80g/L	56
Albumin	35-50g/L	26
Globulin	20-40g/L	30
Human Immunodeficiency Virus Screen	Non-reactive	Non-reactive
Anti-neutrophil Cytoplasmic Antibodies	Negative	Negative
Treponemal Screen	Negative	Negative
Coronavirus	Not Detected	Not Detected
Influenza A	Not Detected	Detected

This gentleman initially received treatment for suspected meningoencephalitis due to symptoms of fever, headache, and weakness. However, cerebral imaging and lumbar puncture (LP) results returned negative. Although GBS is a known potential complication of influenza, neither the LP results nor the clinical examination provided evidence to support this diagnosis.

The patient received supportive management like rest, hydration, and some pain management along with the administration of oseltamivir for five days. Additionally, intravenous antibiotics and antivirals were prescribed to address the suspected meningoencephalitis; however, these therapeutic measures were subsequently terminated.

The patient was hospitalised for a total of 11 days due to peripheral neuropathy, during which significant recovery occurred over the last five days, leading to a discharge with an MRC grading of 4.5/5, indicating only mild subjective weakness and normalised reflexes. At a one-month follow-up, the patient reported complete recovery with no residual weakness.

## Discussion

Influenza is a highly contagious viral infection that predominantly targets the upper and lower respiratory tracts, spreading primarily through airborne droplets. While it is well known for its respiratory symptoms, influenza can also lead to a range of neurological complications, including cognitive impairment, encephalopathy, and seizures [1,2] which highlight its broader impact on health beyond respiratory issues. Some research has suggested a potential link between influenza infection or vaccination and the development of GBS [3,4], a serious autoimmune disorder characterised by rapid onset muscle weakness due to the immune system attacking the peripheral nerves. The pathogenesis of GBS [5] is often attributed to molecular mimicry, where antibodies triggered by an infection mistakenly target nerve antigens. While infections preceding GBS are common, the exact infectious agent is unknown in about 60% of cases, and similar mechanisms may be involved in influenza infections increasing the risk of developing GBS.

Another related condition is mono-neuritis multiplex [6], a type of peripheral neuropathy that occurs due to damage at multiple sites along peripheral nerves. It can arise from various causes, including vasculitis, connective tissue diseases, infections, diabetes, and amyloidosis. In the presented case, the patient experienced multiple nerve involvements without any significant past medical history, making the clinical picture particularly intriguing. Extensive evaluations ruled out common causes such as vasculitis, autoimmune disorders, and infections, with the only significant finding being a positive PCR test for influenza A alongside respiratory symptoms. Remarkably, the patient’s recovery did not necessitate aggressive treatments like corticosteroids; instead, conservative management focused on supportive care, such as rest, hydration, and mild pain management, was sufficient for improvement. This case supports the notion that the neurological symptoms were likely secondary to the influenza A infection rather than indicative of an underlying autoimmune or inflammatory disorder. Nevertheless, establishing a definitive causal relationship between influenza infection and mononeuritis multiplex will require further investigation and additional data to clarify this association and better understand the mechanisms at play.

## Conclusions

Influenza A, a viral infection primarily affecting the respiratory tract, can sometimes have less common neurological effects, including peripheral neuropathy. This condition involves damage to the peripheral nerves, which are responsible for motor and sensory functions throughout the body. In some cases, influenza A can lead to asymmetric weakness-meaning that one side of the body might be more affected than the other. This weakness, along with other symptoms, is linked to damage to specific nerves or nerve groups, which can occur at various sites in the peripheral nervous system. One distinctive feature of influenza A-related peripheral neuropathy is the pattern of recovery. Unlike many other causes of peripheral neuropathy, such as vasculitis (inflammation of the blood vessels) or connective tissue disorders, influenza A-related neuropathy often resolves spontaneously and relatively quickly. Other forms of peripheral neuropathy generally require extended recovery periods, sometimes lasting several months, and they often necessitate treatment with steroids to manage inflammation and promote nerve healing along with extensive physiotherapy. In contrast, influenza A-related neuropathy can improve without the need for such medications.

In this particular case, the patient experienced peripheral neuropathy with asymmetric weakness but did not require steroids or other aggressive treatments to recover. The symptoms improved with conservative management alone, which includes supportive care such as rest, hydration, and some pain management, without the need for immune-suppressing drugs like steroids. This rapid, full recovery aligns with the pattern associated with influenza A, suggesting that the peripheral neuropathy was likely a result of the influenza infection itself rather than another underlying autoimmune or inflammatory condition. Hence, further studies and research are needed to confirm the association of influenza Infection with polyneuropathy. 
